# Maternal Positioning in the Second Stage of Labour and its Relationship with Perineal Trauma: A Systematic Review and Network Meta-Analysis

**DOI:** 10.1007/s00192-025-06512-4

**Published:** 2026-01-22

**Authors:** Estíbaliz Laderas-Díaz, Julián Rodríguez-Almagro, Sandra Martínez-Rodríguez, Rafael Picón-Rodríguez, Antonio Hernández-Martínez

**Affiliations:** 1 Department of Obstetrics & Gynaecology, General Hospital, Ciudad Real, Spain; 2 Department of Nursing, Physiotherapy and Occupational Therapy, Faculty of Nursing, University of Castilla La-Mancha, Ciudad Real, Spain; 3 Department of General Surgery, Santa Barbara Hospital, Puertollano, Spain; 4 Instituto de Investigación Sanitaria de Castilla-La Mancha (IDISCAM), Toledo, Spain

**Keywords:** Maternal position, Perineal trauma, Network, Perineal tear and episiotomy

## Abstract

**Introduction and Hypothesis:**

Perineal trauma is frequent after childbirth and may be influenced by maternal position. We aimed to synthesize pooled estimates comparing positions in the second stage of labour and their association with perineal tears and episiotomy, using a network meta-analysis (NMA) of randomized controlled trials (RCTs).

**Methods:**

Systematic review and NMA of RCTs identified in Cochrane Library Plus, EMBASE, Scopus, PubMed, and ClinicalTrials through June 2024, without language or date restrictions. Outcomes were 1st-degree tears (involving skin and mucosa), 2nd-degree tears (involving perineal muscles), and 3rd/4th-degree tears (involving the anal sphincter and rectal mucosa), as well as episiotomy. We estimated pooled odds ratios (ORs) with 95% confidence intervals (CIs) for upright vs supine/lithotomy and performed a network of individual positions. This review article is based exclusively on previously published literature. Therefore, ethical approval was not required and was exempted by the Institutional Review Board.

**Results:**

Nine RCTs (*n* = 7621) were included. In pooled analyses of upright vs supine/lithotomy, we found no significant differences for 1st-degree tears (OR 1.14, 95% CI 0.76–1.72), 2nd-degree tears (OR 0.98, 95% CI 0.70–1.38), 3rd/4th-degree tears (OR 0.92, 95% CI 0.06–13.93), or episiotomy (OR 0.54, 95% CI 0.22–1.34). In position-level network analyses, standing was ranked lowest-risk for 1st-degree tears (SUCRA 79.1%); squatting ranked lowest-risk for 2nd-degree (SUCRA 93.9%) and for 3rd/4th-degree tears (SUCRA 94.3%); and hands and knees ranked lowest-risk for episiotomy (SUCRA 100%). These position-level findings were based on a limited number of contributing trials and should be interpreted cautiously.

**Conclusions:**

Overall, pooled RCT evidence shows no significant difference in perineal tears of any degree or episiotomy between upright and supine/lithotomy positions. Network estimates suggest potential differences between specific positions, but the evidence is limited and heterogeneous.

Birthing position should primarily reflect women’s preferences and comfort, while considering other key predictors of perineal trauma (e.g. parity, operative delivery, and episiotomy itself). In addition, the findings from this meta-analysis can be used to provide individualized counselling, taking into account each woman’s specific risk factors. This approach may help inform and guide shared decision-making, supporting women in choosing the birthing position that best aligns with their circumstances, preferences, and clinical profile.

**Supplementary Information:**

The online version contains supplementary material available at 10.1007/s00192-025-06512-4.

## Introduction

Vaginal births are often associated with perineal tears of varying degrees. Approximately 80–85% of women are estimated to experience perineal trauma during childbirth [1, 2]. These tears are classified according to severity: first-degree (involving only skin and mucosa), second-degree (involving perineal muscles), third-degree (involving the anal sphincter) and fourth-degree (involving the rectal mucosa), with third- and fourth-degree tears being the most severe and associated with serious problems. First- and second-degree tears, while generally resulting in fewer severe long-term complications, are the most frequent types of perineal trauma and therefore represent a substantial proportion of cases encountered in clinical practice [1, 3].

Perineal trauma can occur spontaneously or as a result of episiotomy [2, 4].

Many factors influence the decision to perform an episiotomy, maternal position may be one of them, either due to technical difficulty in performing the procedure in certain positions or because different positions physiologically modify perineal and fetal dynamics, which may sometimes make episiotomy necessary [5].

These traumas carry significant short- and long-term morbidity. Some associated problems include pain, suture dehiscence, dyspareunia at 6 months postpartum, stress urinary incontinence and even fecal incontinence [1, 2, 4].

The importance of different factors that can influence the appearance of these lesions has been studied. Some are intrinsic to the mother or the new-born, such as maternal age, ethnicity, parity or neonatal birth weight, on which we cannot act. However, various procedures, such as the use of warm compresses, perineal massage or the choice of a specific maternal position, have been described by professionals (midwives and gynaecologists) to minimize the impact of perineal trauma during the second stage of labour [6–8].

Regarding the latter, different maternal positions during the second stage of labour can influence the risk and severity of perineal injury through several physiological mechanisms. Positions such as hands and knees may help redistribute superficial tissue stretch, potentially reducing minor tears, while squatting can enlarge the pelvic outlet and align fetal descent, which may decrease the risk of second-degree or higher tears. In contrast, positions that concentrate pressure on the posterior perineum, such as sitting, may increase strain on perineal muscles and the pelvic floor. Understanding these mechanisms is essential for interpreting clinical outcomes and guiding individualized decisions regarding maternal positioning during labour [9–12].

To date, all published studies have compared positions by grouping them in vertical positions versus horizontal positions or have identified what benefits some positions have compared to others by means of two-group comparisons [9, 13–16]. Currently, there is no systematic review that specifically identifies the risk of perineal trauma by comparing all types of positions via network methodology with indirect comparisons.

Therefore, the main objective of this systematic review and meta-analysis was to compare the different maternal positions during the second stage of labour and to evaluate their effects on the maternal perineum using a network analysis. In doing so, this study aims to clarify the existing evidence so that healthcare professionals can apply it in their routine clinical practice and women can make informed decisions based on scientifically supported information.

## Materials and Methods

This systematic review and meta-analysis was prepared in accordance with the PRISMA statement (Preferred Reporting Items for Systematic Review and Meta-Analyses) [17].

### Search Strategy and Information Sources

A systematic search was carried out in different databases (Cochrane Library Plus, EMBASE, Scopus, PubMed and ClinicalTrials), and the search strategy was adapted for each of them. The search strategy used in PubMed was “Episiotomy” [Mesh] OR “Wounds and Injuries” [Mesh] OR “Lacerations” [Mesh] OR tear * OR trauma. The specific search strategy for each database is detailed in Supplementary Table 1.

### Eligibility Criteria and Outcome Measures

Randomized controlled trials (RCTs) without time or language restrictions were selected. Two reviewers independently (AHM and ELD) evaluated the articles obtained in the bibliographic search by title and abstract in the first stage and subsequently evaluated the selected full texts. Disagreements were resolved by consensus, and if the disagreement continued, a third reviewer assessed the articles (JRA).

The inclusion criteria of the studies were as follows: (I) randomized controlled trials; (II) pregnant woman during the second stage of labour, regardless of the choice of anaesthesia according to randomization; (III) comparison of maternal positions listed in the types of positions; and (IV) inclusion of at least some of the main results.

The exclusion criteria were crossover trials and a lack of sufficient data to calculate the effect size. Trials were included in the abstract form.

The main comparison was the use of any upright position during the second stage of labour compared with the use of the supine or lithotomy position. The secondary comparisons for the network were between any of the positions.

We unified the different terms that referred to the same position to make a simpler classification when performing the analysis. The classification obtained is as follows:

Supine positions: 1. Dorsal (lying on the back); 3. Semi-recumbent (trunk inclined forwards up to 30° with respect to the horizontal); 4. Lithotomy position; 5. Trendelenburg position (head lower than pelvis).

Vertical positions: 1. Sitting; 2. Kneeling; 3. Squat. 4 Standing; 5. Quadrupeds.

The main results of the study were as follows: D perineal strain first degree or type I (no/yes), second degree tear or type II (no/yes), third/fourth degree tear or type III–IV (no/yes), and episiotomy (no/yes).

Outcome definitions: 1st-degree (type I) tear: injury to vaginal mucosa and/or perineal skin only; 2nd-degree (type II): injury extending into perineal muscles but not the anal sphincter; 3rd-degree (type III): injury involving the external and/or internal anal sphincter; 4th-degree (type IV): injury involving the anal sphincter complex and anal epithelium. Where trial-specific definitions were reported, we used them and harmonized terminology to types I–IV. When not explicitly stated, we assumed standard obstetric definitions used in contemporary guidelines.

Criteria for diagnosing perineal lacerations: we accepted the classification reported by each RCT. Most trials used clinical examination immediately after birth by the attending clinician; we extracted the highest-degree tear recorded. Diagnostic criteria were not uniformly detailed across studies.

### Data Extraction and Assessment of Risk of Bias

Data extraction and quality assessment were performed independently by three reviewers. The assessment of risk of bias was carried out using the criteria specified in the Cochrane Handbook for Systematic Reviews of Interventions [18]. Any disagreement was resolved through discussion.

The RCTs were evaluated via the Excel tool of the revised Cochrane *Library Risk of Bias 2* (Rob2) [19]. This tool includes the evaluation of five domains: 1. Bias arising from the randomization process; 2. Bias due to deviations from planned interventions; 3. Bias due to lack of outcome data; 4. Bias in the measurement of the result; and 5. Bias in the selection of the reported outcome. The risk of bias in each domain was considered.

For each RCT, one of the following judgements was made: “low” risk of bias, “some concerns” or “high” risk of bias, depending on the information provided by each study.

### Data Synthesis

For categorical results, the odds ratio (OR) and its 95% confidence interval (95% CI) were used. The Mantel–Haenszel fixed effect model and the Der Simonian–Laird random effects model were used when heterogeneity was found between the studies by means of the I^2^ test and Cochran’s Q statistic. I^2^ values < 25%, 25–50 and > 50% usually correspond to low, medium and high heterogeneity, respectively [20, 21]. Publication bias was also assessed via Egger’s asymmetry test and a funnel plot [22].

The STATA “networkplot” function was used to create a network that describes and presents the different perineal protection interventions. The ordering (ranking) of the different methods is based on the calculation of the areas under the cumulative rank probability curve (SUCRA: surface under the cumulative ranking curve), which is expressed as a percentage and can be interpreted as the probability that the rest of the interventions are lower [23].

Rationale for network meta-analysis: Unlike conventional pairwise meta-analysis, the NMA allows simultaneous comparison and ranking of multiple positions by combining direct and indirect evidence. SUCRA values summarize the probability that each position is among the lowest-risk options for each outcome.

Calculations were made with Stata statistical software (StataCorp. 2017, release 15, College Station, TX) (IBM Corp. 2023, IBM SPSS Statistics for Windows, version 29.0.2.0 Armonk, NY).

#### Ethical/Institutional Review Board Approval

This review article is based exclusively on previously published literature. Therefore, ethical approval was not required and was exempted by the Institutional Review Board.

## Results

### Study Selection

**Fig. 1:**
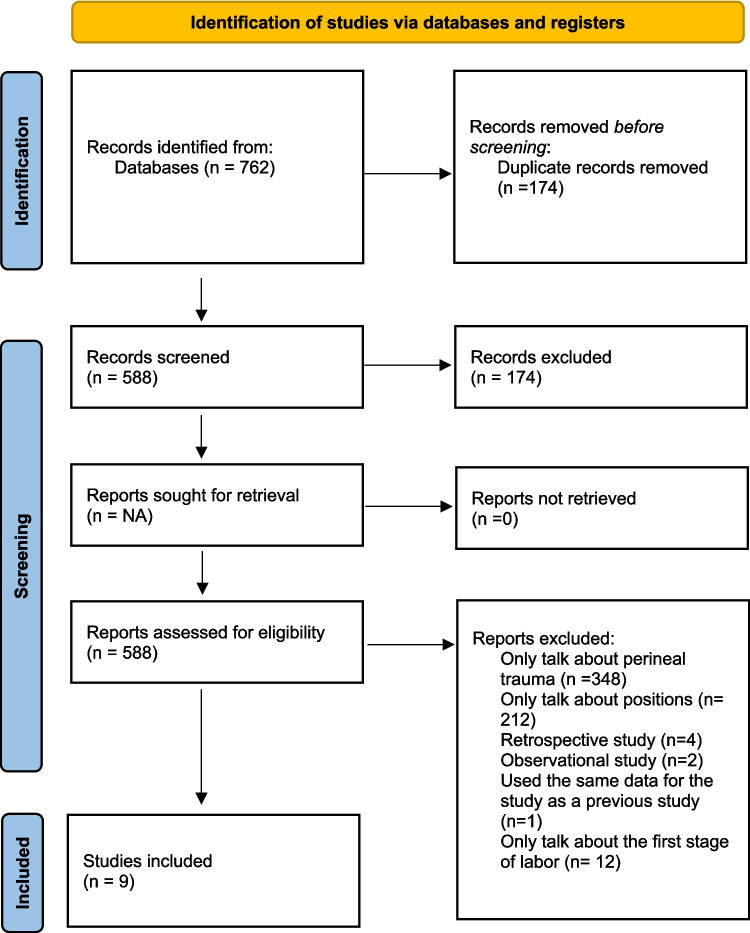
Preferred Reporting Items for Systematic Reviews and Meta-Analyses (PRISMA) 2020 flow diagram for the systematic review

In the bibliographic search, a total of 762 studies were obtained. After elimination of duplicate articles, the titles and abstracts of the remaining 588 documents were selected. After applying the inclusion and exclusion criteria, nine articles were selected for quantitative analysis (meta-analysis) (Fig. 1).

### Study Characteristics

All studies included in the review were RCTs. The sample included 7621 women.

The included studies were conducted in the United Kingdom [24, 25], Ireland [26], Brazil [27], Pakistan [28], Mexico [29], Italy [30], China [31] and India [32].

The sample size of the studies ranged from 127 [27] to 3236 women [24]. In all the trials, the women were in the second stage of labour. The positions evaluated were “vertical or upright” in four RCTs [24, 25, 29, 30], “squatting or sitting” in four RCTs [26–28, 32] and “quadruped” in one RCT [31]. In all cases, they were compared against the “supine position or lithotomy” position.

Most trials permitted women to change position during the second stage [24, 26, 27, 29, 31]. Only a minority quantified crossover [24]; therefore, we analysed outcomes by intention-to-treat where possible. Incomplete reporting of crossover likely attenuates differences between positions.

The number of women in each study, the compared positions, the year, the country and the exclusion criteria of each study are shown in Table 1.

**Table 1:** Characteristics of the included studies

Autor	Year	Compared position N	Horizontal position N	Country	Comparison	Exclusion criteria
*Gardosi J. *[33]	1989	218	209	UK	Upright vs Recumbent	Epidural, elective caesarean section, emergency caesarean section, premature labour, antenatal risk factors, vaginal breech delivery, admission late in labour and miscellaneous reasons
*Crowley P.* [34]	1991	634	596	Ireland	Birth-EZ chair or the conventional delivery bed	Women who had epidural anaesthesia or who were less than 34 weeks pregnant
*Bomfim- Hyppólito S. *[35]	1998	127	121	Brazil	Sitting position vs horizontal position	Newborn lighter than 2500 g or heavier than 4000 g or in which the outcome was other than spontaneous vaginal delivery. Patients on whom episiotomy was judged inevitable
*Nasir A. *[36]	2007	100	100	Pakistan	Squatting position vs lithotomy position	Multiple gestation, malpresentation, previous scar, maternal fever and prenatal diagnosed foetal malformation
*Calvo Aguilar O. *[37]	2013	77	78	México	Upright vs. supine	Patients who had a caesarean section or were not positioned accordingly. Incomplete clinical record or catch sheet
*Serati M. *[38]	2016	296	360	Italy	Upright position vs supine position	The presence of urinary symptoms before delivery, caesarean section or operative vaginal delivery and twin pregnancies
*Zhang H. *[39]	2017	700	700	China	Hands and knees position vs supine position	Pregnancy complications, premature rupture of membranes, non-cephalic presentation, breech position, medical contraindications and/or physical limitations, gestation of less than 37 or over 42 weeks and fluency in Mandarin language sybjuct to the study requirements
*Brocklehurts P. *[40]	2017	1556	1537	UK	Upright position vs lying down position	Consent form missing or incomplete, consent to use data withdrawn, randomised in error, no 2nd stage of labour, epidural not in place at randomization, randomised a‡er delivery
*Vijay Shedmake* *P. *[41]	2021	106	106	India	Squatting position vs lying down position	All those with high- risk pregnancy and those unwilling to participate in the study

### Risk of Bias Assessment of the Quality of the Studies

When the risk of bias analysis was conducted, most of the RCTs were rated as “low risk of bias” for the randomization process, and two trials presented “some concerns”, one of which was due to a lack of information about the process [28] and another due to the voluntary choice of the patients of each position.

Blinding was not possible due to the nature of the intervention, but in most studies, no evidence of deviations from the intended intervention was found. Regarding missing data, all studies were rated as having a “low risk of bias”. The assessment of outcome measurement bias revealed a low risk for most of the measured outcomes, except in one trial [26], which was categorized as having “some concerns” since the beliefs of the evaluators in the beneficial effects of the intervention could influence the reported results. In the evaluation of the selection bias of the reported results, due to the absence of a predefined plan for the analysis, several of the studies were classified as having “some concerns”.

The risk of bias determined with RoB-2 is shown in more detail in Supplementary Figs. 1 and 2.

### Network Meta-Analysis

#### Type I Perineal Tear

**Fig. 2:**
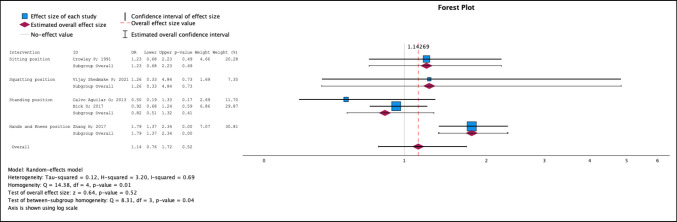
Type I perineal tear. Forest plot

Five studies [24, 26, 29, 31, 32] in which the sitting, squatting, standing, and hands and knees positions were compared with the supine position were included. No significant differences were observed between the vertical and horizontal positions [OR 1.14; 95% CI 0.76–1.72]. In the subgroup analysis, a greater risk of type I perineal tears was observed in the hands and knees, with an OR of 1.79 (95% CI 1.37–2.34), than in the supine position (Fig. 2). The only position that acted as a protective factor, although not significant, was the standing position, with an OR of 0.82 (95% CI 0.51–1.32). The heterogenicity index (I^2^) value was 0.69.

After the network was analysed, the highest SUCRA value was obtained for the standing position, at 79.1%. The order of the positions according to the network analysis from lowest to highest risk was as follows for type I tears: standing, supine, squatting, sitting and hands and knees (Supplementary Figs. 3, 4, 5 and 6).

#### Type II Perineal Tear

**Fig. 3:**
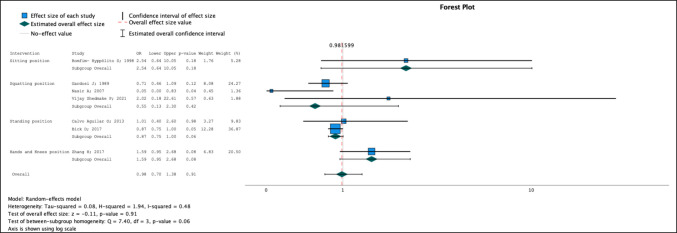
Type II perineal tear. Forest plot

For this purpose, seven studies were included [24, 25, 27–29, 31, 32], in which the sitting, squatting, standing, and hands and knees positions were compared with the supine position. No significant differences were detected between the vertical and horizontal positions [OR 0.98; 95% CI 0.70–1.38]. With respect to the subgroup analysis, no significant differences were observed with respect to type II tears at any of the evaluated positions (Fig. 3). The heterogenicity index (I^2^) value was 0.48. The position with the lowest risk of type II tears, which was not significant, was the squatting position [OR = 0.55; 95% CI 0.13–2.30].

Network analysis revealed that the position with the lowest risk of type II tear was the squatting position, with a SUCRA value of 93.9%. The order of the positions according to the network analysis from lowest to highest risk was squatting, standing, supine, hands and knees, and sitting (Supplementary Figs. 7, 8, 9 and 10).

#### Type III–IV Perineal Tear

**Fig. 4:**
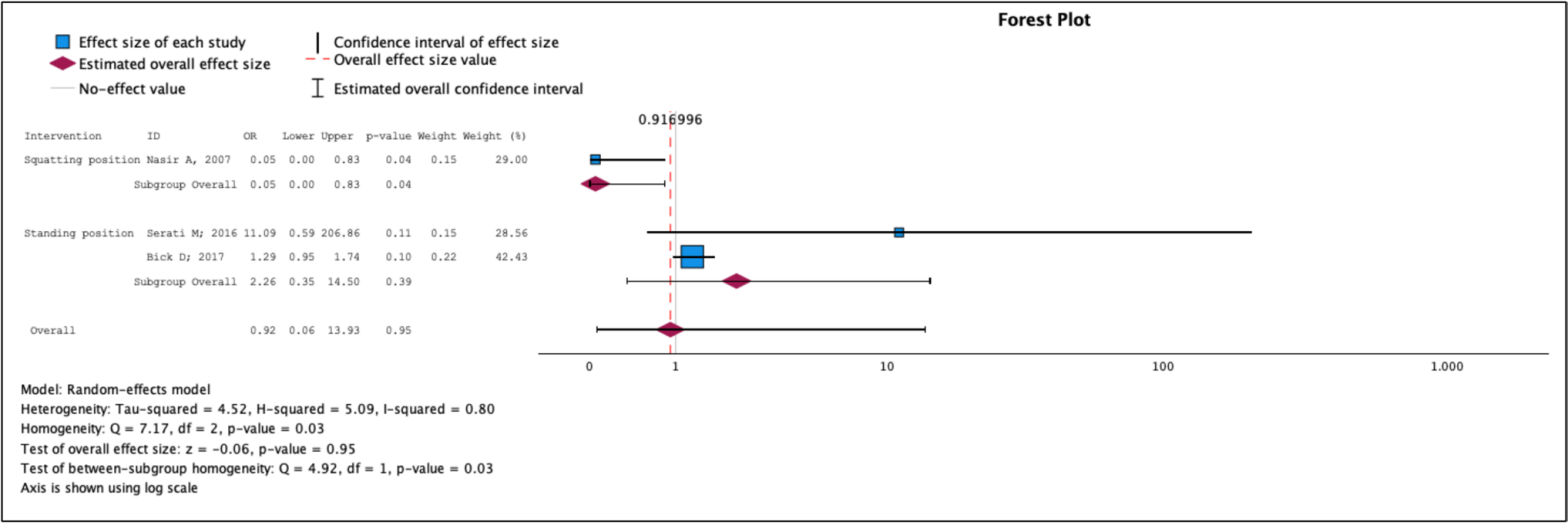
Type III–IV perineal tear. Forest plot

Three studies [24, 28, 30] in which the squatting and standing positions were compared with the supine position were included. No significant differences were detected between the vertical and horizontal positions [OR 0.92; 95% CI 0.06–13.93]. The subgroup analysis revealed that the squat position was the only position that reduced the risk of type III–IV tears (*p* = 0.04) compared with the supine position, with an OR of 0.05 (95% CI 0.00–0.83) (Fig. 4). The heterogenicity index (I^2^) presented a value of 0.80. The position with the lowest risk of type III–IV tear after performing the network was the squatting position, with a SUCRA value of 94.3%. The order of the positions according to the network analysis from lowest to highest risk was squatting, supine and standing (Supplementary Figs. 11, 12, 13 and 14).

### Episiotomy

**Fig. 5:**
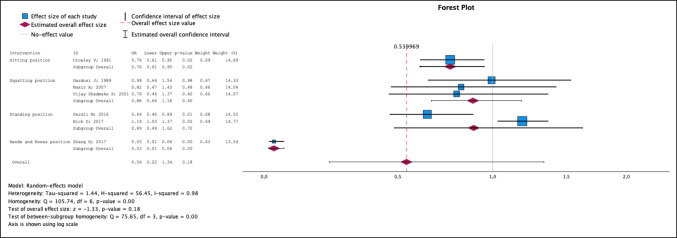
Episiotomy. Forest plot

For this analysis, seven studies [24–26, 28, 30–32] in which the sitting, squatting, standing, and hands and knees versus the supine position were compared were included. No significant differences were observed between the vertical and horizontal positions [OR 0.54; 95% CI 0.22–1.34] (Fig. 5). With respect to the subgroup analysis, significant differences were observed between the sitting position (*p* = 0.02) and the hands and knees (*p* < 0.001) compared with the supine position. The two protective positions brake to the supine position. The hands and knees positions were the most protective, with an OR of 0.03 (95% CI 0.01–0.06). The heterogenicity index (I^2^) value was 0.98. In network analysis, the position with the lowest risk of episiotomy was the hands and knees, with a SUCRA value of 100%. The order of the positions according to the network analysis from lowest to highest risk was hands and knees, sitting, squatting, standing and supine (Supplementary Figs. 15, 16, 17 and 18).

## Discussion

### Main Findings

In our study, we evaluated the relationships between different maternal positions during childbirth and perineal trauma. When the vertical positions were compared with the horizontal positions, no significant differences were detected. However, in the subanalysis by position versus the supine position, we found differences in the risk of perineal trauma. Specifically, for type I tears, the hands and knees position presented a greater risk, whereas the standing position was a protective factor. For type II tears, the position with the lowest risk was squatting, and the position with the highest risk was sitting. For types III–IV, squatting was a protective position, with standing being the position that presented the greatest risk. Finally, for episiotomy, the sitting and the hands and knees positions were protective compared with the supine position, which was ranked as the position of greatest risk.

### Interpretation

Positions during childbirth are increasingly being studied in the field of obstetrics because of their possible influence on the progression of childbirth. There is some consensus that vertical positions seem to facilitate labour, the main justification being the effect of gravity that favours foetal descent. In addition, the lithotomy or supine position has been identified as a favourable position for the progression of labour because it presents an upwards curvature that hinders the descent of the foetus [9].

Currently, there are no systematic reviews in which analyse birthing positions were analysed in relation to perineal trauma. Those that exist thus far have reported two-group analyses in which two types of positions [8, 42] were compared or analyses in which position were grouped into verticals versus horizontals [9, 16].

In a systematic review, 22 studies in which the objective was to evaluate upright positions in the second stage of labour and their importance with regard to the maternal-foetal outcome were selected. With respect to the female perineum, upright positions were shown to be effective in significantly reducing the prevalence of episiotomy [43]. This finding is consistent with the results of our study.

In the systematic review with meta-analysis by Rocha [9], eight studies with a high level of evidence according to the GRADE system were included, and the objective was to evaluate whether vertical positions prevented perineal trauma compared with lithotomy positions. In this review, no statistically significant differences were obtained between the vertical and horizontal positions in the prevention of perineal trauma, as was the case in our analysis. However, in our study, when subanalyses were performed individually for each type of position, the results showed that the supine position seemed to have a greater risk for episiotomy. These results are consistent with those of other studies [9, 16, 24, 27, 43–46].

On the other hand, we have observed variability when classifying the types of perineal trauma. The focus of some studies is on the type I perineal tear, whereas that of other studies is whether or not the perineum is intact, without mention of the type of perineal tear.

This fact has a negative effect on the methodology of our study. In addition, we have identified other factors that may influence our results, such as the fact that some studies have included women with epidural analgesia, whereas others have not.

Episiotomy feasibility versus causality: Lower episiotomy rates with upright positions, especially hands and knees, may partly reflect technical feasibility (greater difficulty performing episiotomy) rather than tissue-level protection [47].

Plausible physiologic mechanisms: Position alters pelvic outlet dimensions, soft-tissue stretch and fetal head alignment. Hands and knees may redistribute superficial stretch (increasing minor tears) without affecting deeper muscle disruption; squatting may enlarge the outlet and align descent, potentially reducing second-degree or higher tears; sitting may concentrate pressure on the posterior perineum. Heterogeneity in analgesia, pushing and perineal support limits causal inference [10, 11, 47–49].

Clinical context: The influence of position appears smaller than established predictors such as nulliparity and operative vaginal delivery [50]. Counselling should integrate these factors when individualizing recommendations.

Clinical guidance should integrate these factors when individualizing recommendations and support women’s choice of birthing position according to their preferences and comfort.

The available evidence is limited and heterogeneous, and results should be interpreted with caution.

### Strengths and Limitations

This study is an update of the research performed to date, but the main strength of this research is that it is the first to compare the different positions in childbirth (network analysis) and not only some of them in isolation, thus obtaining an order of risk of each position for each type of perineal trauma.

The limitations of this research include the lack of standardization of positions during childbirth. Another limitation is the lack of RCTs that study the lateral position, so it has not been possible to include it in this review. On the other hand, the scarcity of RCTs in which women with or without epidural analgesia are studied separately for different positions has prevented us from performing a subanalysis based on this variable, which could be very decisive.

Except for one trial [24], most of the included studies did not specify standardized clinical indications for performing episiotomy, which could explain part of the variability observed in the reported rates.

Notably, the quadruped position is difficult to categorize since, depending on the inclination of the mother, it can be considered vertical or horizontal.

Finally, some of the positions analysed, such as quadruped or squatting, are difficult positions to maintain, so it is difficult to know if the woman maintained that position throughout the second stage of labour or if she alternated between several positions, which would imply bias in the study. Moreover, the precision of the pooled estimates was limited by the small number of included trials and the wide confidence intervals observed in several analyses. Finally, we must be cautious with the interpretation of the results since the number of included trials was small for both the meta-analysis and the network analysis.

### Implications for Practice and Research

The findings of our meta-analysis have important clinical implications with regard to guiding the interventions performed by professionals during birth, as well as helping women make decisions based on the latest evidence. With respect to the field of research, in our bibliographic search, many positions, such as the lateral position, have not yet been studied in terms of their influence on different tears. Similarly, more trials are needed to analyse the effects of epidural surgery on different maternal positions.

## Conclusion

Overall, pooled evidence from randomized controlled trials (RCTs) shows no significant difference in perineal tears of any degree or episiotomy between upright and supine/lithotomy positions. Network estimates suggest potential differences between specific positions, although the evidence is limited and heterogeneous. Our meta-analysis and network analysis revealed that the maternal position with the lowest risk of a type I tear is standing; for type II tears, it is squatting; for type III–IV tears, also squatting; and for episiotomy, it is hands and knees.

Therefore, the choice of birthing position should prioritize women’s preferences and comfort while considering other important predictors of perineal trauma, such as parity, operative delivery and episiotomy. On the basis of these results, women can be guided to make informed decisions, taking into account their individual risk factors and how they may benefit from maternal positioning for perineal protection. This evidence allows pregnant women, in consultation with the professionals caring for them, to make more informed decisions about their position in the second stage of labour.

## Supplementary Information

Below is the link to the electronic supplementary material.

Supplementary file1 (DOCX 197103 KB)

## Data Availability

The datasets generated and analyzed during the current study are available from the corresponding author upon reasonable request.

## References

[1] Goh R, Goh D, Ellepola H. Perineal tears - a review. Aust J Gen Pract. 2018J;47(1–2):35–8. 29429318 10.31128/AFP-09-17-4333

[2] Man R, Morton VH, Morris RK. Childbirth-related perineal trauma and its complications: prevalence, risk factors and management. Obstet Gynaecol Reprod Med. 2024;34(9):252–9.

[3] Folch M, Parés D, Castillo M, Carreras R. Aspectos prácticos en el manejo de las lesiones obstétricas perineales de tercer y cuarto grado para minimizar el riesgo de incontinencia fecal. Cir Esp. 2009;85(6):341–7. 19298954 10.1016/j.ciresp.2008.12.001

[4] Ramar CN, Vadakekut ES, Grimes WR. Perineal lacerations. Treasure Island: StatPearls; 2024. 32644494

[5] Wu LC, Lie D, Malhotra R, Allen JC, Tay JSL, Tan TC, et al. What factors influence midwives’ decision to perform or avoid episiotomies? A focus group study. Midwifery. 2013;29(8):943–9. 23453700 10.1016/j.midw.2012.11.017

[6] Abdelhakim AM, Eldesouky E, Elmagd IA, Mohammed A, Farag EA, Mohammed AE, et al. Antenatal perineal massage benefits in reducing perineal trauma and postpartum morbidities: a systematic review and meta-analysis of randomized controlled trials. Int Urogynecol J. 2020;31(9):1735–45. 10.1007/s00192-020-04302-8. 32399905 10.1007/s00192-020-04302-8

[7] Pierce-Williams RAM, Saccone G, Berghella V. Hands-on versus hands-off techniques for the prevention of perineal trauma during vaginal delivery: a systematic review and meta-analysis of randomized controlled trials. J Matern Fetal Neonatal Med. 2021;34(6):993–1001. 31092083 10.1080/14767058.2019.1619686

[8] Pergialiotis V, Bellos I, Fanaki M, Vrachnis N, Doumouchtsis SK. Risk factors for severe perineal trauma during childbirth: an updated meta-analysis. Eur J Obstet Gynecol Reprod Biol. 2020;247:94–100. 32087423 10.1016/j.ejogrb.2020.02.025

[9] Rocha BDda, Zamberlan C, Pivetta HMF, Santos BZ, Antunes BS. Upright positions in childbirth and the prevention of perineal lacerations: a systematic review and meta-analysis. Rev Esc Enferm. 2020;54:1–11. 10.1590/S1980-220X2018027503610 32935765

[10] Desseauve D, Fradet L, Lacouture P, Pierre F. Position for labor and birth: state of knowledge and biomechanical perspectives. Eur J Obstet Gynecol Reprod Biol. 2017;208:46–54. 27888706 10.1016/j.ejogrb.2016.11.006

[11] Borges M, Moura R, Oliveira D, Parente M, Mascarenhas T, Natal R. Effect of the birthing position on its evolution from a biomechanical point of view. Comput Methods Programs Biomed. 2021;200. 10.1016/j.cmpb.2020.105921 33422852

[12] Havelková L, Krofta L, Kochová P, Liška V, Kališ V, Feyereisl J. Persistent occiput posterior position and stress distribution in levator ani muscle during vaginal delivery computed by a finite element model. Int Urogynecol J. 2020(7). 10.1007/s00192-019-03997-8. 10.1007/s00192-019-03997-8 PMC7306020 31197428

[13] Gupta JK, Hofmeyr GJSR. Position in the second stage of labour for women without epidural anaesthesia. Cochrane Database Syst Rev. 2004;1:58. 10.1002/14651858.CD002006.pub3 22592681

[14] Kemp E, Kingswood CJ, Kibuka M, Thornton JG. Position in the second stage of labour for women with epidural anaesthesia. Cochrane Database System Rev. 2013;2013(1). 10.1002/14651858.CD008070.pub2 23440824

[15] Berta M, Lindgren H, Christensson K, Mekonnen S, Adefris M. Effect of maternal birth positions on duration of second stage of labor: systematic review and meta-analysis. BMC Pregnancy Childbirth. 2019;19(1):1–8. 31801479 10.1186/s12884-019-2620-0 PMC6894325

[16] Roberts CL, Algert CS, Cameron CA, Torvaldsen S. A meta-analysis of upright positions in the second stage to reduce instrumental deliveries in women with epidural analgesia. Acta Obstet Gynecol Scand. 2005;84(8):794–8. 16026407 10.1111/j.0001-6349.2005.00786.x

[17] Liberati A, Altman DG, Tetzlaff J, Mulrow C, Gøtzsche PC, Ioannidis JPA, et al. The PRISMA statement for reporting systematic reviews and meta-analyses of studies that evaluate healthcare interventions: explanation and elaboration. BMJ. 2009;339. 10.1136/bmj.b2700 PMC2714672 19622552

[18] Higgins JPT, Green S. Manual Cochrane de revisiones sistemáticas de intervenciones. Cochrane. 2011;(March):1–639.

[19] Sterne JAC, Savović J, Page MJ, Elbers RG, Blencowe NS, Boutron I, Cates CJ, Cheng H-Y, Corbett MS, Eldridge SM, Hernán MA, Hopewell S, Hróbjartsson A, Junqueira DR, Jüni P, Kirkham JJ, Lasserson T, Li T, McAleenan A, Reeves BC, Shepperd S, Shrier I, Stew HJ. The Cochrane Collaboration’s tool for assessing risk of bias in randomised trials. 2019;366.

[20] Higgins JPT, Thompson SG. Quantifying heterogeneity in a meta-analysis. Stat Med. 2002;21(11):1539–58. 12111919 10.1002/sim.1186

[21] Higgins JPT, Thompson SG, Deeks JJ, Altman DG. Measuring inconsistency in meta-analyses. BMJ. 2003;327(7414):557–60. 12958120 10.1136/bmj.327.7414.557 PMC192859

[22] Egger M, Smith GD. Bias in location and selection of studies. BMJ. 1998;316(7124):61–6. 9451274 10.1136/bmj.316.7124.61 PMC2665334

[23] Salanti G, Ades AE, Ioannidis JPA. Graphical methods and numerical summaries for presenting results from multiple-treatment meta-analysis: an overview and tutorial. J Clin Epidemiol. 2011;64(2):163–71. 20688472 10.1016/j.jclinepi.2010.03.016

[24] Bick D, Briley A, Brocklehurst P, Hardy P, Juszczak E, Lynch L, et al. Upright versus lying down position in second stage of labour in nulliparous women with low dose epidural: BUMPES randomised controlled trial. BMJ. 2017;359. 10.1136/bmj.j4471 PMC5646262 29046273

[25] Gardosi J, Hutson N, B-Lynch C. Randomised, controlled trial of squatting in the second stage of labour. Lancet. 1989;334(8654):74–7. 10.1016/s0140-6736(89)90315-2 2567873

[26] Crowley P, Elbourne D, Ashurst H, Garcia J, Murphy D, Duignan N. Delivery in an obstetric birth chair: a randomized controlled trial. Br J Obstet Gynaecol. 1991;98(7):667–74. 1883790 10.1111/j.1471-0528.1991.tb13453.x

[27] Bomfim-Hyppólito S. Influence of the position of the mother at delivery over some maternal and neonatal outcomes. Int J Gynecol Obstet. 1998;63(Suppl. 1). 10.1016/s0020-7292(98)00186-6 10075214

[28] Nasir A, Korejo R, Noorani KJ. Child birth in squatting position. J Pak Med Assoc. 2007;57(1):19–22. 17319414

[29] Aguilar OC, Romero ALF, García VEM. Comparison of obstetric and perinatal outcomes in childbirth upright posture vs. supine [Comparación de resultados obstétricos y perinatales del parto en postura vertical versus supina]. Ginecol Obstet Mex. 2013;81(1):1–10. 23513398

[30] Serati M, Di Dedda MC, Bogani G, Sorice P, Cromi A, Uccella S, et al. Position in the second stage of labour and de novo onset of post-partum urinary incontinence. Int Urogynecol J. 2016;27(2):281–6. 10.1007/s00192-015-2829-z 26337426

[31] Zhang H, Huang S, Guo X, Zhao N, Lu Y, Chen M, et al. A randomised controlled trial in comparing maternal and neonatal outcomes between hands and knees delivery position and supine position in China. Midwifery. 2017;1(50):117–24. 10.1016/j.midw.2017.03.022 28414983

[32] Shedmake PV, Wakode SR. A hospital-based randomized controlled trial—comparing the outcome of normal delivery between squatting and lying down positions during labour. J Obstet Gynaecol India. 2021;71(4):393–8. 34566298 10.1007/s13224-021-01439-4 PMC8418581

[33] Gardosi J, Hutson N, B-Lynch C. Randomised, controlled trial of squatting in the second stage of labour. Lancet. 1989;334(8654):74–7. 10.1016/s0140-6736(89)90315-2 2567873

[34] Crowley P, Elbourne D, Ashurst H, Garcia J, Murphy D, Duignan N. Delivery in an obstetric birth chair: a randomized controlled trial. Br J Obstet Gynaecol. 1991;98(7):667–74. 1883790 10.1111/j.1471-0528.1991.tb13453.x

[35] Bomfim-Hyppólito S. Influence of the position of the mother at delivery over some maternal and neonatal outcomes. Int J Gynecol Obstet. 1998;63(Suppl. 1). 10.1016/s0020-7292(98)00186-6 10075214

[36] Nasir A, Korejo R, Noorani KJ. Child birth in squatting position. J Pak Med Assoc. 2007;57(1):19–22. 17319414

[37] Aguilar OC, Romero ALF, García VEM. Comparación de resultados obstétricos y perinatales del parto en postura vertical versus supina. Ginecol Obstet Mex. 2013;81(1):1–10. 23513398

[38] Serati M, Di Dedda MC, Bogani G, Sorice P, Cromi A, Uccella S, et al. Position in the second stage of labour and de novo onset of post-partum urinary incontinence. Int Urogynecol J. 2016;27(2):281–6. 26337426 10.1007/s00192-015-2829-z

[39] Zhang H, Huang S, Guo X, Zhao N, Lu Y, Chen M, et al. A randomised controlled trial in comparing maternal and neonatal outcomes between hands-and-knees delivery position and supine position in China. Midwifery. 2017;1(50):117–24. 10.1016/j.midw.2017.03.022 28414983

[40] Bick D, Briley A, Brocklehurst P, Hardy P, Juszczak E, Lynch L. Upright versus lying down position in second stage of labour in nulliparous women with low dose epidural: BUMPES randomised controlled trial. BMJ. 2017; 10.1136/bmj.j4471 10.1136/bmj.j4471 PMC5646262 29046273

[41] Shedmake PV, Wakode SR. A hospital-based randomized controlled trial—comparing the outcome of normal delivery between squatting and lying down positions during labour. J Obstet Gynecol India. 2021;71(4):393–8. 34566298 10.1007/s13224-021-01439-4 PMC8418581

[42] Jones K, Webb S, Manresa M, Hodgetts-Morton V, Morris RK. The incidence of wound infection and dehiscence following childbirth-related perineal trauma: a systematic review of the evidence. Eur J Obstet Gynecol Reprod Biol. 2019;240:1–8. 31202973 10.1016/j.ejogrb.2019.05.038

[43] Deliktas A, Kukulu K. A meta-analysis of the effect on maternal health of upright positions during the second stage of labour, without routine epidural analgesia. J Adv Nurs. 2018;74(2):263–78. 28881046 10.1111/jan.13447

[44] Peppe MV, Stefanello J, Infante BF, Kobayashi MT, De Oliveira BC, Brito LGO. Perineal trauma in a low-risk maternity with high prevalence of upright position during the second stage of labor. Rev Bras Ginecol Obstet. 2018;40(7):379–83. 30016809 10.1055/s-0038-1666810

[45] Bodner-Adler B, Bodner K, Kimberger O, Lozanov P, Husslein P, Mayerhofer K. Women’s position during labour: influence on maternal and neonatal outcome. Wien Klin Wochenschr. 2003;115(19–20):720–3. 14650948 10.1007/BF03040889

[46] Familiari A, Neri C, Passananti E, Marco G Di, Felici F, Ranieri E, et al. Maternal position during the second stage of labor and maternal-neonatal outcomes in nulliparous women: a retrospective cohort study. AJOG global reports. 2023;3(1). 10.1016/j.xagr.2023.100160 PMC9941360 36825260

[47] Satone PD, Tayade SA. Alternative birthing positions compared to the conventional position in the second stage of labor: a review. Cureus. 2023;15(4):e37943. 37223195 10.7759/cureus.37943 PMC10202683

[48] Gorman J, Roberts CA, Newsham S, Bentley GR. Squatting, pelvic morphology and a reconsideration of childbirth difficulties. Evol Med Public Health. 2022;10(1):243. 35663511 10.1093/emph/eoac017 PMC9154243

[49] Kurnaz D, Balacan Z, Karacam Z. The effects of upright positions in the second stage of labor on perineal trauma and infant health: a systematic review and meta-analysis. J Educ Res Nurs. 2022;19(4):383–95. 10.5152/jern.2022.09454.

[50] Kalis V, Laine K, De Leeuw JW, Ismail KM, Tincello DG. Classification of episiotomy: towards a standardisation of terminology. BJOG. 2012;119(5):522–6. 22304364 10.1111/j.1471-0528.2011.03268.x

