# Supplementary material for: Maternal Positioning in the Second Stage of Labour and its Relationship with Perineal Trauma: A Systematic Review and Network Meta-Analysis
Source: Int Urogynecol J. 2026 Jan 22;37(5):1167–77. doi: 10.1007/s00192-025-06512-4 (PMC13226342; doi:10.1007/s00192-025-06512-4)
Supplement: Supplementary file 1 — Supplementary file1 (DOCX 197103 KB) [file 192_2025_6512_MOESM1_ESM.docx]

**Supplementary table 1. *Search Strategies****.*

| **Data Base** | **Search Strategies** | **Hits** |
| --- | --- | --- |
| ***Pubmed*** | ("Episiotomy"[MeSH Terms] OR "Wounds and Injuries"[MeSH Terms] OR "Lacerations"[MeSH Terms] OR "tear*"[All Fields] OR ("injuries"[MeSH Subheading] OR "injuries"[All Fields] OR "trauma"[All Fields] OR "Wounds and Injuries"[MeSH Terms] OR ("wounds"[All Fields] AND "injuries"[All Fields]) OR "Wounds and Injuries"[All Fields] OR "trauma s"[All Fields] OR "traumas"[All Fields])) AND "labor stage, second"[MeSH Terms] | 346 |
| ***Scopus*** | (("episiotomy" OR "wounds and injuries" OR "lacerations" OR “tear” OR "trauma" ) AND ( position ) AND  ( labor AND stage AND second ) ) | 232 |
| ***Clinical Trials*** | position \| ( EXPAND[Concept] "episiotomy" OR EXPAND[Concept] "wounds and injuries" OR EXPAND[Concept] "lacerations" OR EXPAND[Concept] "tear" OR EXPAND[Concept] "trauma" ) AND ( labor AND stage AND second ) | 87 |
| ***Cochrane Library Plus*** | (("episiotomy" OR "wounds and injuries" OR "lacerations" OR "tear" OR "trauma") AND (position) AND (labor AND stage AND second) ) | 97 |

**Supplementary figure 1.** ***Summary of risk of bias assessments as percentages across included studies according to RoB 2 (Version 2 of the Cochrane risk-of-bias tool for randomized trials).***

As percentage (intention-to-treat)

|  | Low risk | Some concerns | High risk |
| --- | --- | --- | --- |
| Randomization process | 77.8 | 22.2 | 0 |
| Deviations from intended interventions | 77.8 | 22.2 | 0 |
| Mising outcome data | 100 | 0 | 0 |
| Measurement of the outcome | 88.9 | 11.1 | 0 |
| Selection of the reported result | 44.4 | 55.6 | 0 |
| Overall Bias | 33.3 | 33.3 | 33.3 |

**Supplementary figure 2. *Summary of risk of bias assessments for each study according to RoB 2 (Version 2 of the Cochrane risk-of-bias tool for randomized trials)***

**Supplementary figure 3. *Classification of interventions (SUCRA) – Tears type I.***

**Supplementary figure 4. *Classification of interventions according to Network analysis. Tears type I.***

**Supplementary figure 5.** ***Funnel Plot – Tears type I.***

**Supplementary figure 6. *Network -Tears type I.***

SIP: Sitting position, KKP: Hands and Knees position;

SQR: Squatting position; STP: Standing Position. CT: Supine Position

**Supplementary figure 7. *Classification of interventions (SUCRA) – Tears type II.***

**Supplementary figure 8. *Classification of interventions according to Network analysis. Tears type II.***

**Supplementary figure 9*. Funnel Plot - Tears type II***

**Supplementary figure 10. *Network – Tears type II.***

SIP: Sitting position, KKP: Hands and Knees position;

SQP: Squatting position; STP: Standing Position. CT: Supine Position

**Supplementary figure 11. *Classification of interventions (SUCRA) – Tears type III-IV.***

**Supplementary figure 12. *Classification of interventions according to Network analysis – Tears type III-IV.***

**Supplementary figure 13. *Funnel Plot – Tears type III-IV***

**Supplementary figure 14.  *Network – Tears type III-IV.***

SQP: Squatting position; STP: Standing Position. CT: Supine Position

**Supplementary figure 15. *Classification of interventions (SUCRA) - Episiotomy.***

**Supplementary figure 16. *Classification of interventions according to Network analysis - Episiotomy.***

**Supplementary figure 17. *Funnel Plot – Episiotomy.***

**Supplementary figure 18. *Network - Episiotomy.***

SIP: Sitting position, KKP: Hands and Knees position;

SQP: Squatting position; STP: Standing Position. CT: Supine Position
